# Mesenchymal stem cell transplantation after acute myocardial infarction: a meta-analysis of clinical trials

**DOI:** 10.1186/s13287-021-02667-1

**Published:** 2021-12-07

**Authors:** Armin Attar, Fateme Bahmanzadegan Jahromi, Shahin Kavousi, Ahmad Monabati, Asma Kazemi

**Affiliations:** 1 Department of Cardiovascular Medicine, TAHA Clinical Trial Group, School of Medicine, Shiraz University of Medical Sciences, Zand Street, Shiraz, Iran; 2 Students’ Research Committee, Shiraz University of Medical Sciences, Shiraz, Iran; 3 Hematology Research Center, Shiraz University of Medical Sciences, Shiraz, Iran; 4 Department of Pathology, Shiraz University of Medical Sciences, Shiraz, Iran; 5 Nutrition Research Center, Shiraz University of Medical Sciences, PO Box 71645-111, Shiraz, Iran

**Keywords:** Meta-analysis, Mesenchymal, Stem cell, Myocardial infarction

## Abstract

**Background:**

Trials investigating the role of mesenchymal stem cells (MSCs) in increasing ejection fraction (LVEF) after acute myocardial infarction (AMI) have raised some controversies. This study was conducted to find whether transplantation of MSCs after AMI can help improve myocardial performance indices or clinical outcomes.

**Methods:**

Randomized trials which evaluated transplantation of MSCs after AMI were enrolled. The primary outcome was LVEF change. We also assessed the role of cell origin, cell number, transplantation time interval after AMI, and route of cell delivery on the primary outcome.

**Results:**

Thirteen trials including 956 patients (468 and 488 in the intervention and control arms) were enrolled. After excluding the biased data, LVEF was significantly increased compared to the baseline among those who received MSC (WMD = 3.78%, 95% CI: 2.14 to 5.42, *p* < 0.001, *I*^2^ = 90.2%) with more pronounced effect if the transplantation occurred within the first week after AMI (MD = 5.74%, 95%CI: 4.297 to 7.183; *I*^2^ = 79.2% *p* < 0.001). The efficacy of trans-endocardial injection was similar to that of intracoronary infusion (4% [95%CI: 2.741 to 5.259, *p* < 0.001] vs. 3.565% [95%CI: 1.912 to 5.218, *p* < 0.001], respectively). MSC doses of lower and higher than 10^7^ cells did not improve LVEF differently (5.24% [95%CI: 2.06 to 8.82, *p* = 0.001] vs. 3.19% [95%CI: 0.17 to 6.12, *p* = 0.04], respectively).

**Conclusion:**

Transplantation of MSCs after AMI significantly increases LVEF, showing a higher efficacy if done in the first week. Further clinical studies should be conducted to investigate long-term clinical outcomes such as heart failure and cardiovascular mortality.

**Supplementary Information:**

The online version contains supplementary material available at 10.1186/s13287-021-02667-1.

## Background

Myocardial infarction (MI), a common presentation of coronary artery disease, is the main cause of death in the developed countries [1]. Over the past few decades, a rise in the incidence of heart failure (HF) was observed in contrast to the reduction in the mortality rate after MI [2]. In spite of the current guideline-directed therapy [3], mortality and morbidity of post-MI heart failure are quite high [4]. Although the current managements for HF are prolonging the patients’ life while improving their symptoms, they do not restore the normal histologic architecture and induce regeneration in the damaged cells. Therefore, improving confirmed treatments and developing further approaches to treat patients with post-MI heart failure are strongly required [5].

One approach to restore the damaged myocardium after MI has pointed at stem cell-based therapies [6]. Cell therapy was initially proposed a putative approach to reconstruct the damaged myocardium. Cell therapy moved forward to human studies with outstanding speed, using skeletal myoblasts in patients with HF in 2001 [7], and BM-derived mononuclear cell (BM-MNC) transplantation in acute MI in 2002 [8]. Since then, many studies on animals and humans have been performed to assess different cell types and their ability to repair cardiac and vascular damage in the settings of MI, cardiomyopathy, etc. Most of the studies on cell therapy in AMI are done by BM-MNCs. Based on a meta-analysis by Fisher and colleagues, treatment with BM-MNC would increase LVEF after AMI by 2.72% [9]. However, studies on the mesenchymal stem cells (MSC)s are more encouraging. In the TAC-HFT trial, it was shown that MSC was about twice as much effective as the bone marrow-derived mononuclear cells (BM-MNCs) [10].

Mesenchymal stem cells (MSCs) are a population of cells initially isolated from the BM and have been found in other organs and tissues such as Wharton’s jelly and adipose tissue [11]. The International Society for Cellular therapy describes Mesenchymal stem cells (MSCs) as a population of cells that adheres to plastic in standard culture conditions, expresses CD73, CD90, and CD105 in the absence of CD34, CD45, HLA-DR, CD14 or CD11b, CD79a, or CD19 surface molecules, and has the ability to differentiate into osteoblasts, adipocytes, and chondroblasts in vitro [12]. Due to the availability of these resources, these tissues are becoming the dominant source for isolation of MSC for clinical uses [13]. Furthermore, the safety of MSCs-therapy from these origins has been confirmed previously [13]. Because of their desirable features, such as an easily accessible source of adult stem cell with multi-lineage potential, simplicity of isolation from bone marrow and expansion, maintenance of stem cell niches, potential of allogeneic transplant, recruitment of endogenous stem cells, and secretion of paracrine factors, MSCs are progressively used in clinical trials of stem cell therapy [14]. Clinical trials using MSCs in AMI are controversial but encouraging. Some have shown promising results while others have been neutral. In addition, most of them are conducted by a low sample volume. Furthermore, many questions such as the optimal number of stem cells that must be injected and the optimal time course of delivery to maximize recovery of cardiac function post-infarct remain to be answered. Consequently, performing a meta-analysis seemed essential to reveal the real effect of these cells, so this study aimed to investigate the effect of MSC on the cardiac function after AMI and factors affecting it by conducting a meta-analysis.

## Method

The Preferred Reporting Items for Systematic reviews and Meta-Analyses (PRISMA) statement (http://www.prisma-statement.org/) was used in this systematic review. The PICOS (participants, intervention, comparison, and outcomes of study design) model was used to formulate the study question.

### Search strategy and study selection criteria

Relevant studies were identified by searching PubMed, Scopus, the Cochrane Library (to April 2021), Embase, Pubmed, Google Scholar, and clinicaltials.gov without language limit. The search terms used included infarction, ischemia, ischemic disease and cardiac, heart, myocardial and mesenchymal stem cell, progenitor, stromal cell, multipotent stromal cell, alone or in combination with each other.

All clinical trials (randomized or non-randomized) which had investigated the effects of stem cell therapy on myocardial function in patients with acute myocardial infarction were eligible to be included in the meta-analysis. Two reviewers independently screened the eligibility of studies. Any discrepancies were resolved by discussion with a third author.

### Risk of bias (quality) assessment

Two authors independently assessed the quality of the studies using Cochrane Risk of Bias Tool for Randomized Controlled Trials; the studies were assessed for criteria such as random sequence generation, allocation concealment, blinding of participants, personnel and outcome assessment, incomplete outcome data, and selective reporting. In cases all the criteria were met, or only had one criterion rated unclear, the quality was judged as good. When one criterion was met or two criteria were unclear, the article was graded as fair quality; in case two or more criteria were not met or unclear, the article was considered to be of poor quality. Any disagreement was resolved by discussion.

### Data extraction

Data extraction was carried out independently by two reviewers. The accuracy of the extracted data was checked by a third reviewer. If the included studies did not provide the required data, we requested the necessary data from corresponding authors. The extracted data included authors, year of publication, manuscript type, study design, cell origin, cell number, route of delivery, number of participants, age, sex, measurement tool, primary intervention, transplantation time after MI, follow-up, diabetes mellitus (DM), Hypertension (HTN), Systolic blood pressure (SBP), diastolic blood pressure (DBP), and heart rate (HR); we also collected data about the mean values and the standard deviation of the following outcomes at baseline, final, and change from baseline: EF, left ventricular end diastolic diameter (LVEDD), left ventricular end systolic diameter (LVESD), hospitalization for CHF, and infarction size.

### Statistical analysis

All the statistical procedures were performed using Stata software version 13 (StataCorp LP, College Station, TX, USA). Mean change from baseline and its standard deviation (SD) for each outcome were extracted. In case the mean change was not reported, we calculated the mean changes and estimated SD using the correlation coefficient for the studies that reported baseline, after intervention, and change values. The weighted mean difference (WMD) and its corresponding SD were calculated using the DerSimonian and Laird method using a random effects model, which considers the between-study variation. The heterogeneity between studies was assessed using the Cochran’s Q test and *I*2. To find the possible sources of heterogeneity, we used meta-regression and subgroup analysis. The variables sex, age (< 65 year vs. ≥ 65 year), cell number, cell origin, time of transplantation, follow-up duration, rout of delivery, measurement tool, and primary intervention were specified for conducting subgroup analysis priory. The nonlinear potential effects for age, transplant time, follow-up duration, cell number, and baseline values were examined using fractional polynomial modeling. However, the number of studies in the subgroups was only sufficient in the subgroup analysis by cell number and time of transplantation. Moreover, we performed the analysis by excluding the studies with high risk of bias studies.

If at least 10 studies were available, we explored potential small-study effects, such as publication bias, using Egger’s test and funnel plots. *p* values ≤ 0.05 were considered statistically significant. Influence analysis was performed to test the possible effect of individual studies on the final results.

## Results

### Study identification and selection

**Fig. 1:**
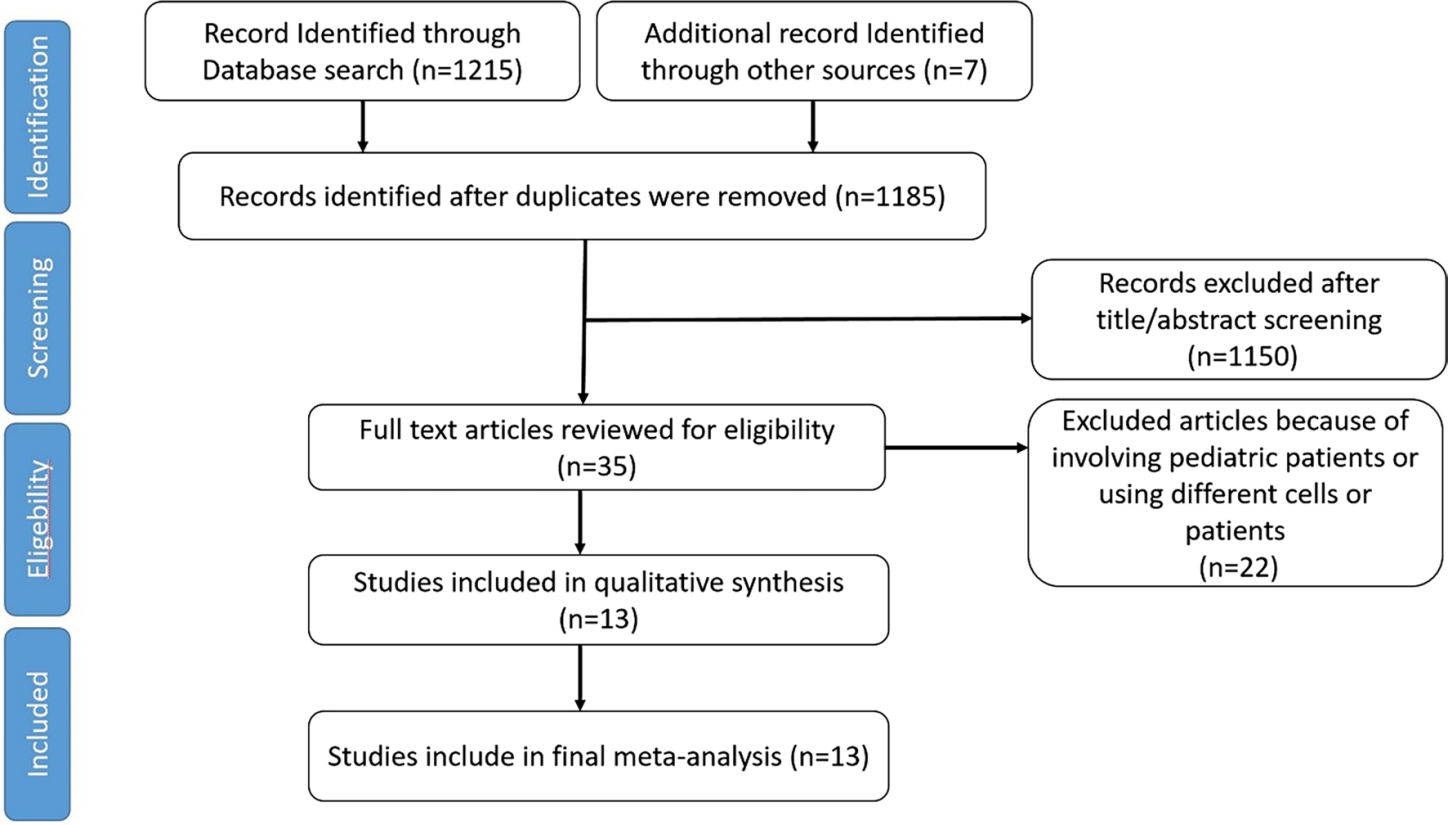
PRISMA flow diagram of the study

Figure 1 shows the PRISMA flow diagram of the study. In the initial search in databases, 1215 articles were identified; also 7 articles were found through other ways of search, of which 1150 references were excluded in the first screening. From 35 remaining articles, 13 studies with 956 patients (468 patients in the intervention arm and 488 in this systematic review and meta-analysis). Characteristics of the included clinical trials on the effect of stem cell therapy on acute myocardial infarction are presented in Table 1. The articles were mostly published from 2010 to 2018 (2003–2021). The baseline LVEF of patients were in the range of 20.2 to 57.2. The mesenchymal stem cells were injected through intracoronary route in ten studies, intramyocardial route in two studies, and intravenous in one study. The total cell numbers injected were between 2.3 and 85 × 10^6^. The mean duration of patients’ follow-up after transplantation was between 6 and 182.6 months. The sample sizes were in the range of 13–390 patients. All studies had performed PCI as the primary intervention, except one that had performed thrombolytic therapy. Twelve studies used echocardiography as a measurement tool, eight studies used SPECT, three used angiography, and just two studies used MRI for measuring ejection fraction (Table 1) [13, 15–26].

**Table 1:** Studies’ characteristics

Author	Year of publication	Participant comorbidity number DM(intervention, control) HTN(intervention, control)	Sample size number (intervention, control)	Age year (intervention, control) Mean (SD)	Gender (intervention, control) Number of females	Cell origin	Route of delivery	Method of measurement	Follow-up duration (month)
Hare	2009	6,1 16,9	34,19	59(12.3) 55.1(10.2)	6,4	BMSC	IV	Echocardiography, MRI	6
Gao	2013	6,5 13,11	21,22	55(1.6) 58.6(2.5)	0,3	BMSC	IC	Echocardiography, SPECT	24
Chen	2004	Not reported	34,35	58(7) 57(5)	2,1	BMSC	IC	Echocardiography, PET	3
Chulikana	2014	Not reported	10,10	47.31(12.1) 47.79(6.48)	0,2	BMSC	IC	Echocardiography, MRI, SPECT	24
Penn	2018	1,2 4,3	19,6	53(9.9) 53(8)	1,1	BMSC	IC	Echocardiography, angiography	
Lee	2014	5,8 14,12	30,28	53.9(10.5) 54.2(7.7)	3,3	BMSC	IC	Echocardiography, SPECT	182.6,179.5
Rodrigo	2013	1,5 4,18	9,45	56(8) 61(11)	2,10	BMSC	IM	Echocardiography, SPECT	54.3
Gao	2015	17,14 33,26	58,58	57.3(103) 56.7(1.7)	3,7	Umblical	IC	Echocardiography, SPECT	18
Wang	2014	Not reported	28,30	56.1(9.8) 58(10.2)	16,9	BMSC	IC	Echocardiography, angiography	6
Kharlamov	2007	45,48,55 131,127,132	131,127,132	58.32(9.12) 59.44(10.26) 57.25(7.68)	8,8,15	autologous, adipose	IM	Echocardiography, angiography, SPECT	12
Hautgraff	2012	Not reported 6,2	9,4	61(2.1) 55(7.5)	2,0	Adipose	IC	SPECT	36
Kim	2018	3,2 5,5	14,12 14,12	56.45 56.45	0,0	BMSC	IC	SPECT Echocardiography	6
Zhang	2021	8,5 13,11	21,22	58.94	1,3	BMSC	IC	Echocardiography	12

### Quality assessment of studies

Quality assessment of studies is presented in Table 2. Among the thirteen studies, one was non-randomized [18] and five studies did not report the method of randomization [17, 19, 20, 22, 24]. Five articles had low risk of allocation concealment [13, 15, 16, 21, 23]. Only seven studies were blinded to the participants or researcher [13, 15, 20, 21, 23, 25, 26]; two studies were not blind [16, 19], and four were unclear [17, 18, 22, 24]. Outcomes blinding for assessors was good in nine articles [13, 15–17, 19–21, 23, 25]. Two studies had incomplete outcome data [15, 19, 23], but the others were at low risk. No article had selective reporting. As a whole, three studies were judged as having good quality [13, 21, 25], five poor [18, 19, 22, 24], and three fair [20, 23, 26] qualities.

**Table 2:** Quality assessment analysis

RCT	Random sequence generation	Allocation concealment	Blinding of participants and personnel	Blinding of outcome assessors	Incomplete outcome data	Selective reporting	
Chen et al. [22]	U	U	U	U	L	L	Poor
Chullikana et al. [21]	L	L	L	L	L	L	Good
Gao et al. [15]	L	L	L	L	H	L	Fair
Gao et al. [16]	L	L	H	L	L	L	Fair
Hare et al. [23]	L	L	L	L	H	L	Fair
Lee et al. [19]	U	U	H	L	H	L	Poor
Wang et al. [17]	U	U	U	L	L	L	Poor
Kharlamov et al. [24]	U	U	U	U	L	L	Poor
Houtgraaf et al. [13]	L	L	L	L	L	L	Good
Rodrigo et al. [18]	H	U	U	U	L	L	Poor
Penn et al. [20]	U	U	L	L	L	L	Fair
Kim et al. [25]	L	U	L	L	L	L	Good
Zhang et al. [26]	L	U	L	U	L	L	Fair

*H*: high risk, *U*: unclear risk, *L*: low risk (Cochrane Handbook For Systematic Reviews Of Intervention)

### LVEF

**Fig. 2:**
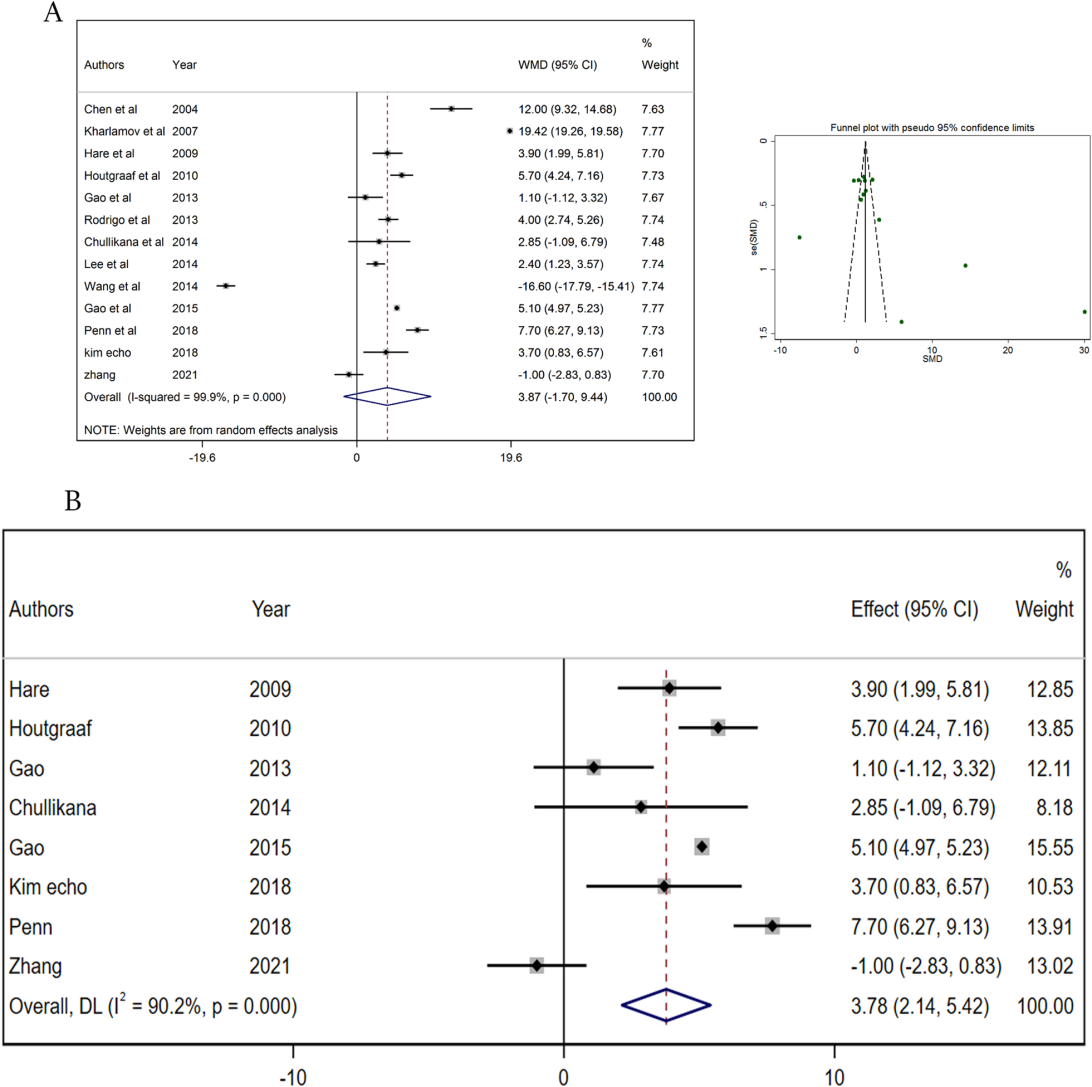
**A** Forest and funnel plot for ejection fraction before excluding the biased studies **B** forest plot for ejection fraction after excluding biased studies *WMD* weighted mean difference *CI* confidence interval

Although pooled analysis of 13 studies (956 patients) revealed no significant change in LVEF (WMD = 3.868%, 95% CI: − 1.699 to 9.435, *p* = 0.173, *I*^2^ = 99.9%), however, excluding studies with poor quality and high risk of bias showed a significant increase (WMD = 3.78%, 95% CI: 2.14 to 5.42, *p* < 0.001, *I*^2^ = 90.2%; Fig. 2).

### Other echocardiographic indices

The observed change in LVEDV that was measured by seven studies was not significant (WMD − 5.076 ml,95% Cl, − 11.909 to 1.757, *p* value = 0.145, *I*^2^ = 96.2%); the reduction in LVEDV remained nonsignificant after excluding the four studies with high risk of bias (WMD =  − 5.985 ml, 95% CI, − 15.907 to 3.938, *p* value = 0.237, *I*^2^ = 95.1%). The change in LVESV, as a pooled analysis of six studies, was not significant (WMD =  − 2.892 ml, 95%Cl, − 6.779 to 0.995, *p* value = 0.145, *I*^2^ = 38.4%) and after excluding the three studies with high risk of bias, the reduction was greater and still marginally nonsignificant (WMD =  − 5.963 ml, 95% CI, − 12.18 to 0.26, *p* value = 0.17, *I*^2^ = 43.8%); (Additional file 1).

### Infarct size

No significant change was observed in the infarct size as a pooled analysis of three studies on 91 patients (WMD =  − 5.12, 95%Cl − 20.73 to 10.50, *p* value = 0.52, *I*^2^ = 86.7). After excluding the results from a study which quality control showed that it has poor quality with high chance of bias, the analysis revealed a significant decrease in the infarct size (WMD = -8.91, 95% Cl − 22.08 to 4.26, *p* value = 0.002, *I*^2^ = 100; Additional file 2).

### Hospitalization for heart failure

Analysis of seven studies with 315 participants indicated that 21 out of 191 patients (10.99%) in the intervention group and 21 out of 167 patients (12.57%) in the control group were hospitalized due to heart failure, which showed no significant therapeutic effect (RR = 0.882, 95%Cl 0.563 to 1.381, *p* value = 0.584). Only one study had a high risk of bias; excluding this study did not have a serious effect on the result (RR = 0.917, 95%Cl 0.580 to 1.450, *p* value = 0.712).

### Subgroup analyses

**Fig. 3:**
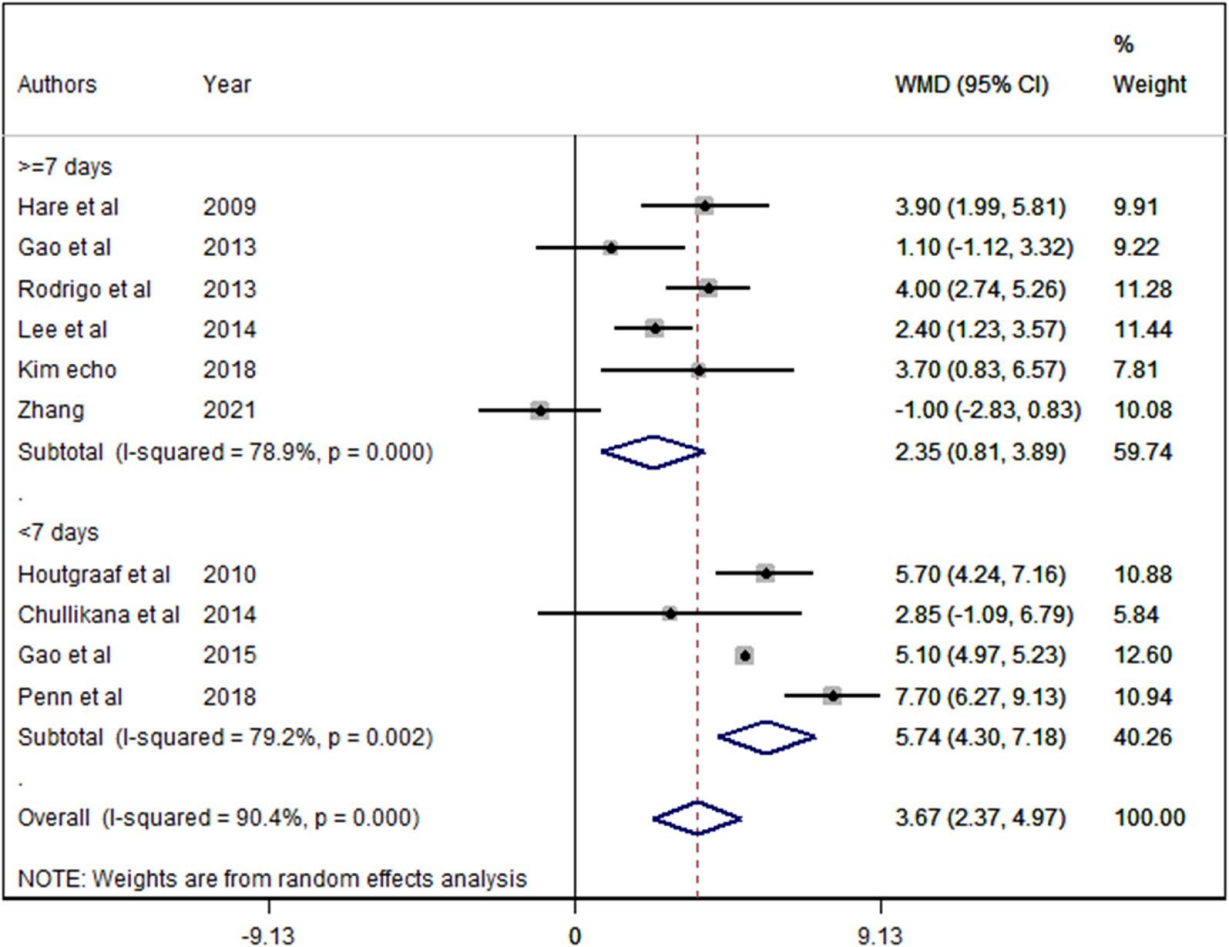
Subgroup analysis for the time interval between AMI and transplantation after excluding the biased studies *WMD* weighted mean difference *CI* confidence interval

Transplantation of MSC within the first week after AMI significantly increased LVEF by 5.740% (95% CI 4.297 to 7.183; *p* value < 0.001; *I*^2^ = 79.2%), but not after 1 week by (WMD = 1.16%, 95% CI − 5.417 to 7.733; *p* value = 0.730; *I*^2^ = 99.2%%, and after exclusion of high risk of bias studies WMD = 1.85%; 95% CI − 0.61 to 4.31; *I*^2^ = 80.7%; *p* = 0.14; Fig. 3).

**Fig. 4:**
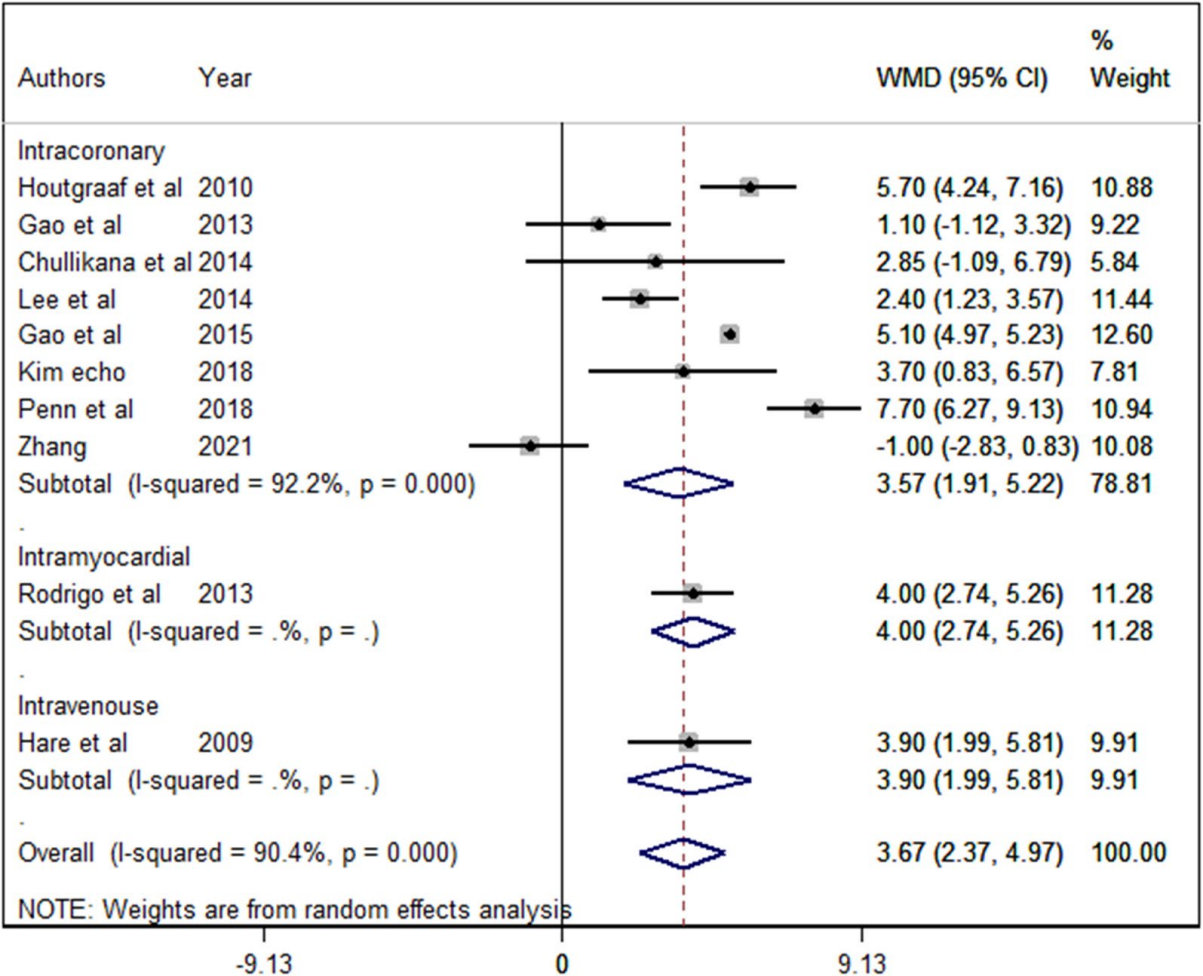
Subgroup analysis for the route of delivery after excluding the biased studies

To perform a subgroup analysis for route of delivery, after excluding poor quality trials biased studies, we found no significant difference between the group effects on EF. Also, it should be noted that in two routes, only one study was performed: Intravenous (WMD 3.90, 95%Cl 1.99 to 5.81, *p* < 0.001), Intramyocardial (Subendocardial) (WMD 4.00, 95%Cl 2.74 to 5.26, *p* < 0.001), and Intracoronary (WMD 3.57, 95%Cl 1.91 to 5.22, *p* < 0.001; Fig. 4).

According to subgroup analysis done for follow-up duration, the efficacy of this new treatment has become more obvious after following the patients for more than 12 months both before and after excluding the biased studies (WMD = 4.21, 95%Cl 2.95 to 5.46, *p* value < 0.001; Additional file 3). Between-group analysis was done, and the difference was significant (*p* between group < 0.001).

Performance of a subgroup analysis for cell source revealed that umbilical cord-derived stem cells had a more positive effect on EF (WMD = 5.1, 95%Cl 4.239 to 7.161, *p* value < 0.001) in comparison with bone marrow derived cells (WMD = 1.98, 95%Cl 1.813 to 5.714, *p* value = 0.498, *I*^2^ = *87.4*) and adipose derived cells (WMD = 5.1, 95%Cl 4.971 to 5.229, *p* value = 0.067; Additional file 4).

Comparison between MSC injections higher or lower than ${10}^{7}$ exhibited no statistically significant LVEF improvement (4.81(95%Cl − 5.37 to 14.99, *p* = 0.354) vs. 2.36(95%Cl − 0.62 to 5.34, *p* = 0.121)). In spite of this finding, after excluding the biased studies, analysis showed that cell injections lower than ${10}^{7}$ could significantly improve LVEF more (4.55[95%Cl 3.92 to 5.17, *p* < 0.001] vs. 5.05]95%Cl 4.92 to 5.18, *p* = 0.121]; Additional file 5).

### Meta-regression analysis

Meta-regression analyses for age (Additional file 6), follow-up duration, transplant time, cell number, and the baseline values did not show a significant trend for LVEF either in presence or after excluding high risk of bias studies. Similarly, when we evaluated dose-responses, there was no evidence for nonlinear dose response relations between the abovementioned variables and LVEF.

### Publication bias and sensitivity analysis

Although a slight asymmetry was seen in the funnel plots (Additional file 7), there was no evidence for publication bias based on Egger’s test (*p* = 0.131).

## Discussion

In the present study, by including 13 RCTs and 956 patients, we found that MSC therapy improved LVEF after AMI by 3.67%; if this therapy was performed within the first week, its effect might increase to 5.74%. Also, when transplantation dose was less than ${10}^{7}$ cells, it might improve LVEF as well. To the best of our knowledge, this meta-analysis is the largest available meta-analysis in the field. Most of available previous studies have included both acute and chronic ischemic diseases [27] or have enrolled animal studies [28] to increase the population number; if they did not do it, the analysis was done with a sample size less than 450 studies and at most of eight trials [29, 30]. Our study is almost twice as large as previous meta-analyses.

Since the introduction of regenerative medicine in cardiology, many clinical trials have been conducted to evaluate various types of cells. Among them, BM-MNCs have been studied widely and most of our knowledge about stem cell therapy in AMI is derived from those studies. We asked questions such as “When should these cells be transplanted?,” “From which route?,” and “in which patients?” in BM-MNCs trials, but not in MSCs. Consequently, performance of meta-analysis for MSCs to answer these questions seemed essential.

Meta-analyses of BM-MNCs trials including those with a patients’ level data have indicated that transplantation of BM-MNC after AMI may improve LVEF by 2.72% [9]. In our study, we noticed an LVEF improvement of 3.67% by transplantation of MSC. This finding is similar to the results of TAC-HFT trial. In that study, it was shown that MSCs were about twice as much effective as the BM-MNCs [10]. Furthermore, in the POSIEDON trial, it was shown that allogenic MSC was as safe and effective as autologous ones [31]. This gives MSCs the potential to be used as off the shelf. These characteristics make MSCs a more attractive and effective source of cell in regenerative cardiology as compared to BM-MNCs.

The role of timing on the effect of stem cell therapy after AMI has been widely investigated in BM-MNCs trials, but not on MSCs. In the Regenerate AMI (transplantation within 24 h) [32], TIME (transplantation within 1 week) [33], and Late TIME (transplantation after 2–3 weeks) [34] trials, the effect of timing on the efficacy of BM-MNCs on LVEF was assessed. Results of the mentioned trials and pool patient data meta-analyses [35, 36] revealed that the best transplantation time after AMI to improve the myocardial function would be 3–7 days after AMI. If it is done sooner, it may cause loss of transplanted stem cells due to the high inflammatory status within the myocardium and later may reduce the effect as myocyte loss and fibrosis will be stablished. No trial has evaluated this issue for MSCs, and our meta-analysis has paved the way in the field.

The effect of the number of intracoronary MSCs transplanted on the myocardial function recovery has been investigated in animal and preclinical studies. In the pig model, Fiarresga et al. have shown that a higher number of intracoronary transplanted MSCs would increase the chance of microvascular obstruction and myocardial injury [37]. A similar finding in sheep was also noticed [38]. Our finding regarding the effect of cell number is in agreement with those of animal studies.

The exact mechanisms accounting for the beneficial effects of using stem cells in AMI in preclinical and clinical studies are not clear. The data supporting the theory on differentiation of the transplanted cells as a mechanism of improvement in the recipient heart are very poor; even if all of the remaining cells are transformed into the cardiomyocytes, it would not be sufficient to account for the useful effects reported [39]. Differentiation of the transplanted cells into new vessels has been observed in different cells, such as MSCs [40], and it has been suggested that vasculogenesis may result in rescuing the cardiomyocytes in the hypoxic area. It is challenging to imagine (consider) how the vasculogenesis mechanism could be a main mechanism in patients who had already successful coronary revascularization after an AMI; however, it is obvious that this phenomenon can be responsible for some of the advantageous effects of cell therapy. Recently, the paradigm has shifted from these mechanisms to the paracrine effect theory, which suggests that most of the beneficial effects after cell therapy are obtained through signals such as cytokines that are released in a paracrine signaling by the injected cells and alter the nearby cells and the recipient heart [41].

Since the efficacy of MSC therapy after AMI seems to be limited, many investigators have tried to enhance the quality of these cells before transplantations. Lin and colleagues suggested that addition of IGF-1 would enhance viability, migration, and anti-apoptosis of MSC in myocardial infarction [42]. Wu and coworkers assessed the role of sFRP2 in this situation [43], and Huang et al. assessed the role of Secreted frizzled-related proteins (Sfrps) [44].

This study had some limitations. The most important one is that three trials had reported results that were significantly incompatible with the findings of other studies. Although the final results of including and excluding them were nearly similar, due to development of a large LVEF change interval, the results would become significant only by excluding them [(WMD = 3.87%, 95% CI: − 1.699 to 9.435, *p* = 0.173) vs. (WMD = 3.673%, 95% CI: 2.374 to 4.973, *p* < 0.001)]. The other issue that should be addressed is that the baseline LVEF enrolled in the trials was not similar; this might have affected the final results. Another important hint is that most of studies used echocardiography as their main method of LVEF assessment. However, cardiac MRI with the potential to show myocardial fibrosis and scar size and mass may be a more accurate way of assessment.

## Conclusion

It can be concluded that transplantation of MSCs after AMI significantly increases LVEF. Performing the operation within the first week after AMI might augment this efficacy. Performance of a large clinical trial in future to see if MSC transplantation can help prevent hospitalization form heart failure after AMI seems to be necessary. The PREVENT-TAHA trial (https://clinicaltrial.gov, NCT05043610.) will help finding the answer.

## Supplementary Information

**Additional file 1**. Forest plot for the left ventricular end diastolic diameter (Top) left ventricular end systolic diameter (Bottom) WMD weighted mean difference CI confidence interval.

**Additional file 2**. Forest plot for infarction size before (top) and after (Botteom) excluding the biased studies.

**Additional file 3**. Subgroup analysis for the follow-up duration.

**Additional file 4**. Subgroup analysis for stem cell resource.

**Additional file 5**. Subgroup analysis for the number of cells transplanted before and after excluding the biased studies.

**Additional file 6**. Dose response analysis for age before (top) and after (Bottom) excluding the biased studies.

**Additional file 7**. Publication bias analysis.

## Data Availability

The original data are available from the corresponding author on request.

## Abbreviations

AMI: Acute myocardial infarction
BM-MNC: Bone marrow derived mononuclear cell
CHF: Congestive heart failure
LVEDD: Left ventricular end diastolic diameter
LVEF: Left ventricular ejection fraction
LVESD: Left ventricular end systolic diameter
MSC: Mesenchymal stem cell
RCT: Randomized controlled trial
