# Supplementary figures and images for: Mesenchymal stem cell transplantation after acute myocardial infarction: a meta-analysis of clinical trials
Source: Stem Cell Res Ther. 2021 Dec 7;12:600. doi: 10.1186/s13287-021-02667-1 (PMC8650261; doi:10.1186/s13287-021-02667-1)

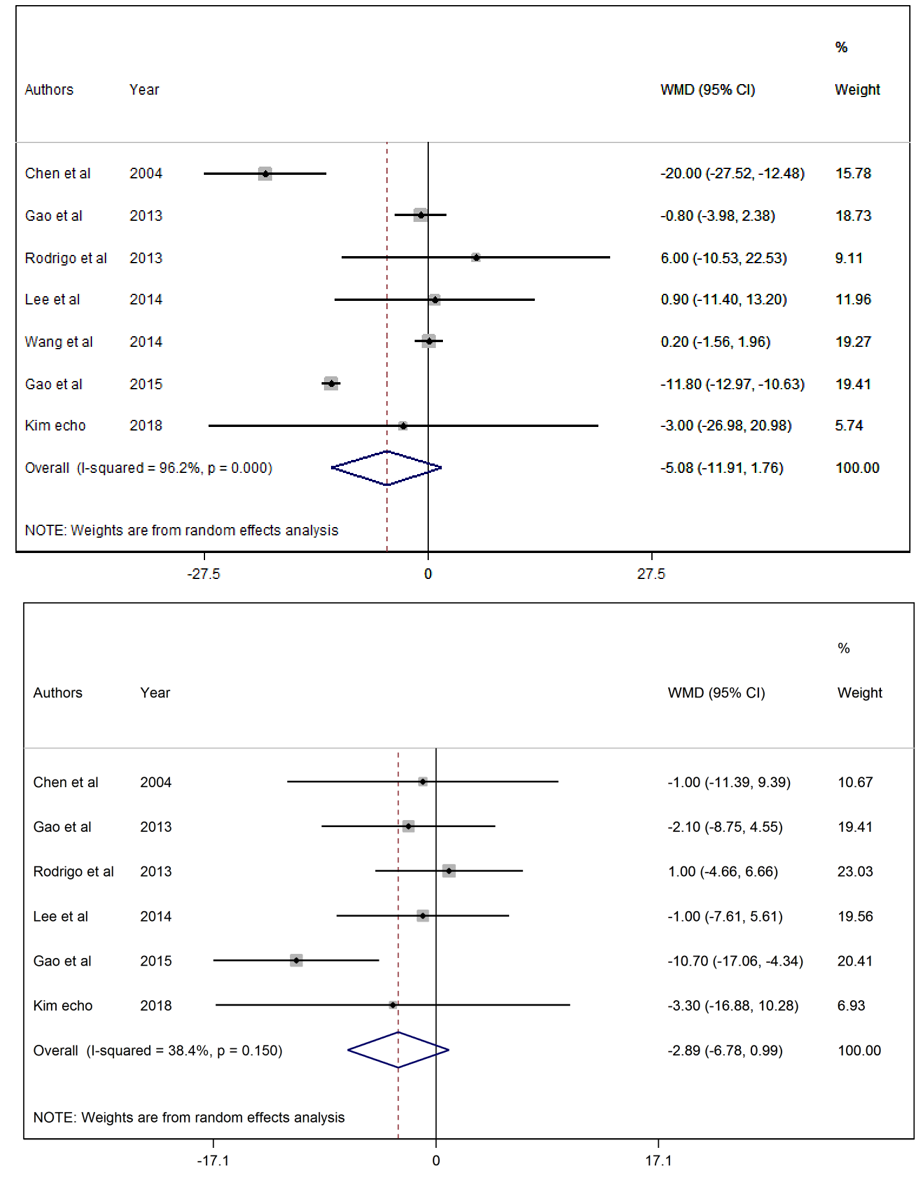

Supplement: Supplementary file 1 — Additional file 1. Forest plot for the left ventricular end diastolic diameter (Top) left ventricular end systolic diameter (Bottom) WMD weighted mean difference CI confidence interval. [file 13287_2021_2667_MOESM1_ESM.tif]

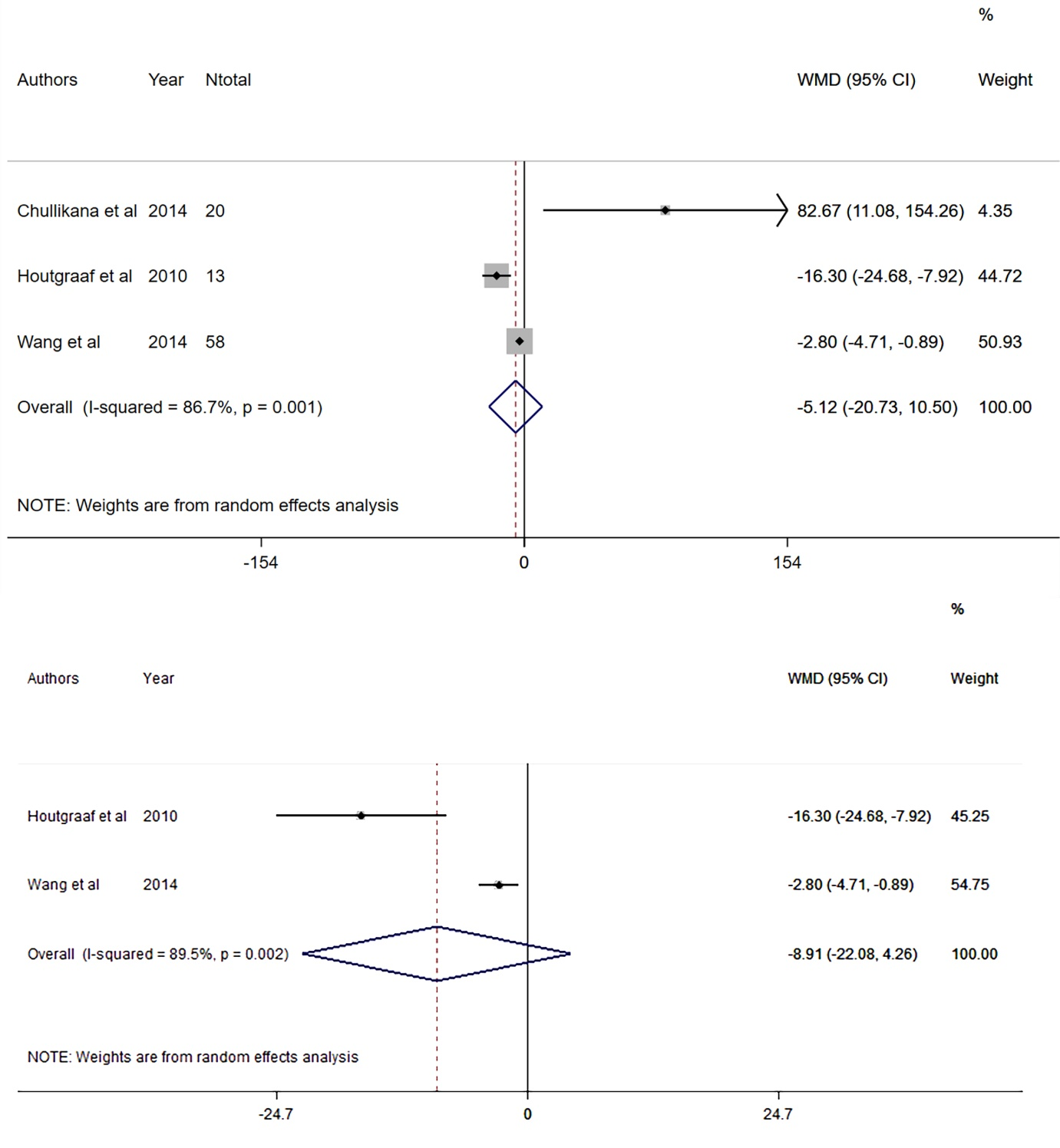

Supplement: Supplementary file 2 — Additional file 2. Forest plot for infarction size before (top) and after (Botteom) excluding the biased studies. [file 13287_2021_2667_MOESM2_ESM.tif]

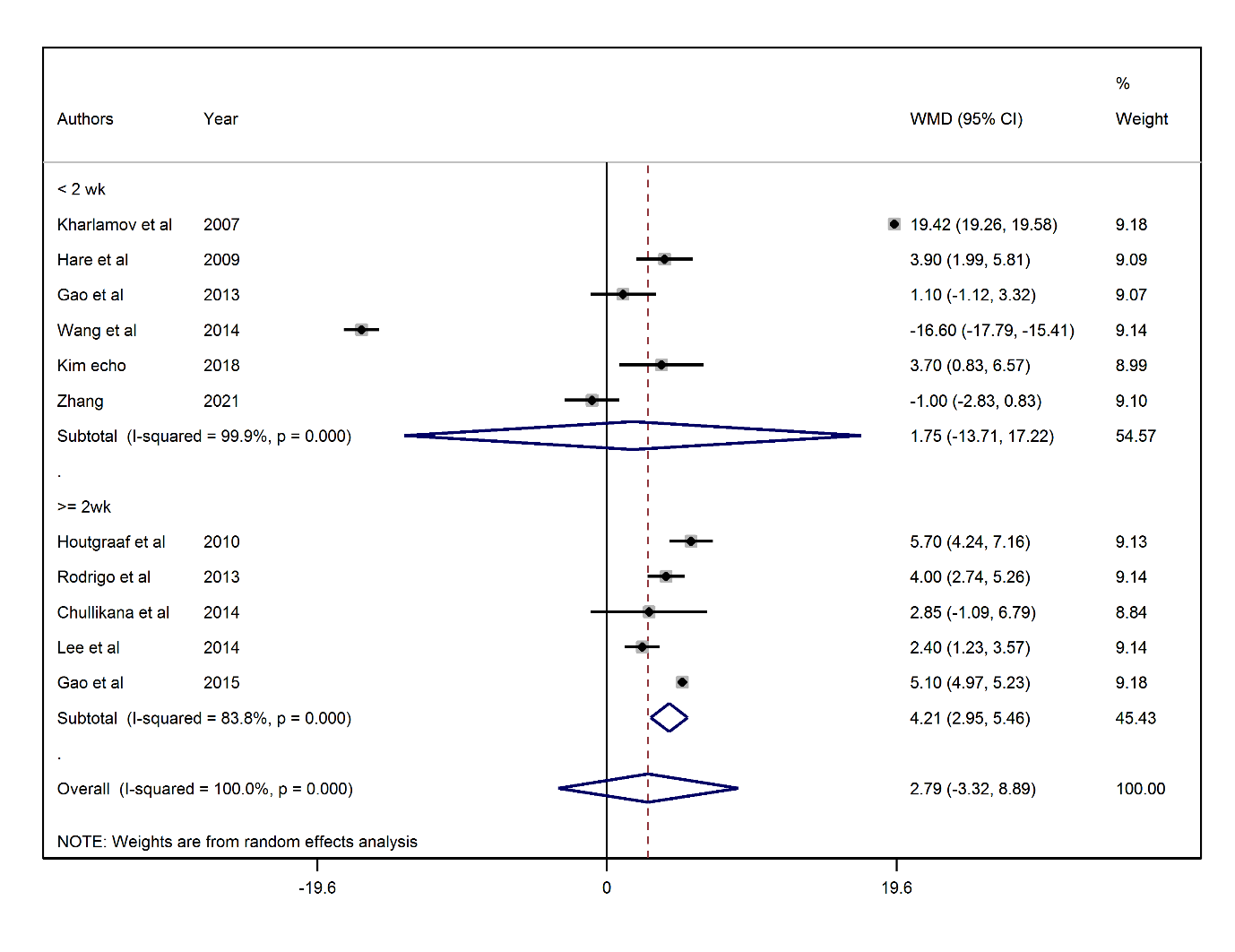

Supplement: Supplementary file 3 — Additional file 3. Subgroup analysis for the follow-up duration. [file 13287_2021_2667_MOESM3_ESM.tif]

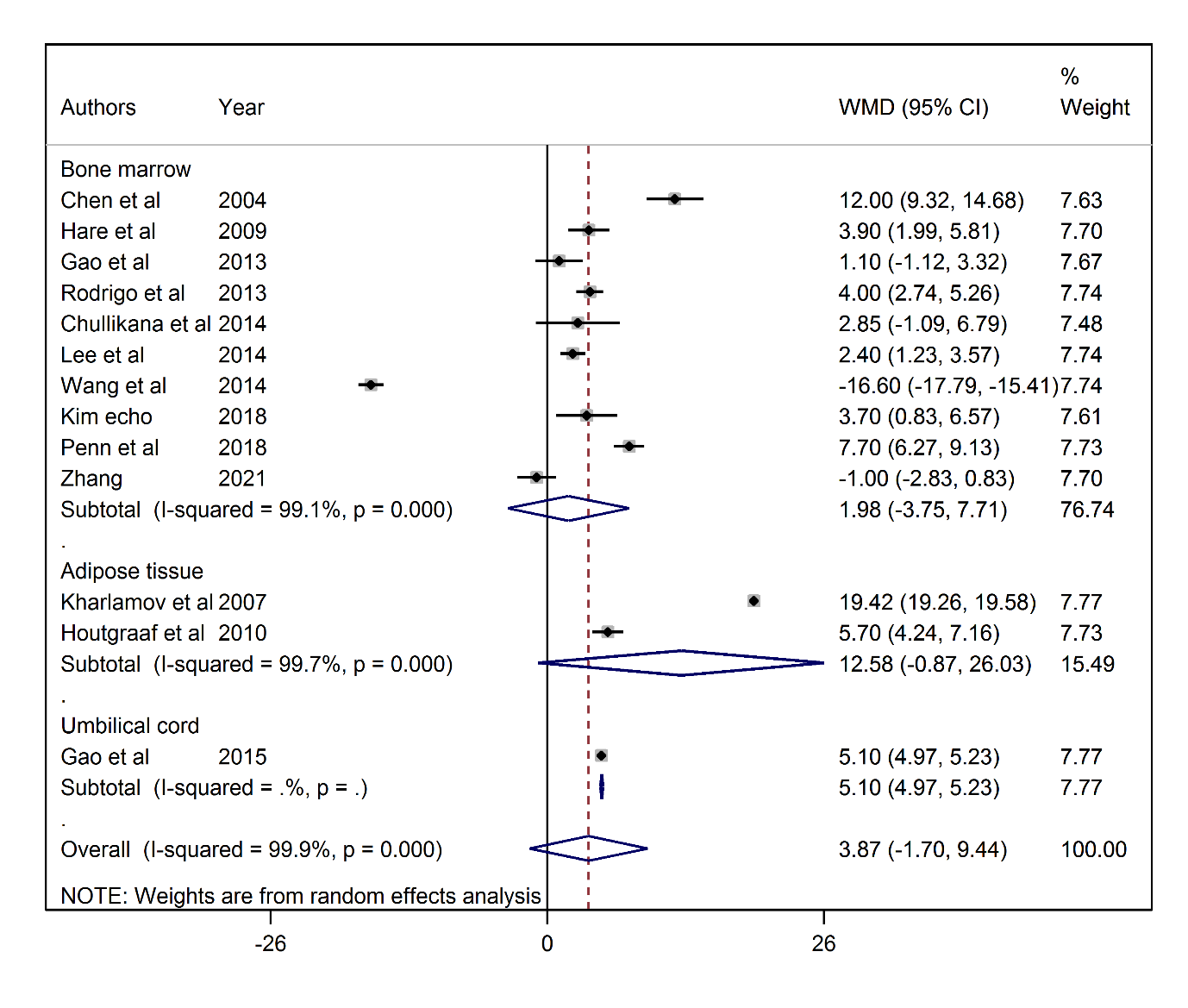

Supplement: Supplementary file 4 — Additional file 4. Subgroup analysis for stem cell resource. [file 13287_2021_2667_MOESM4_ESM.tif]

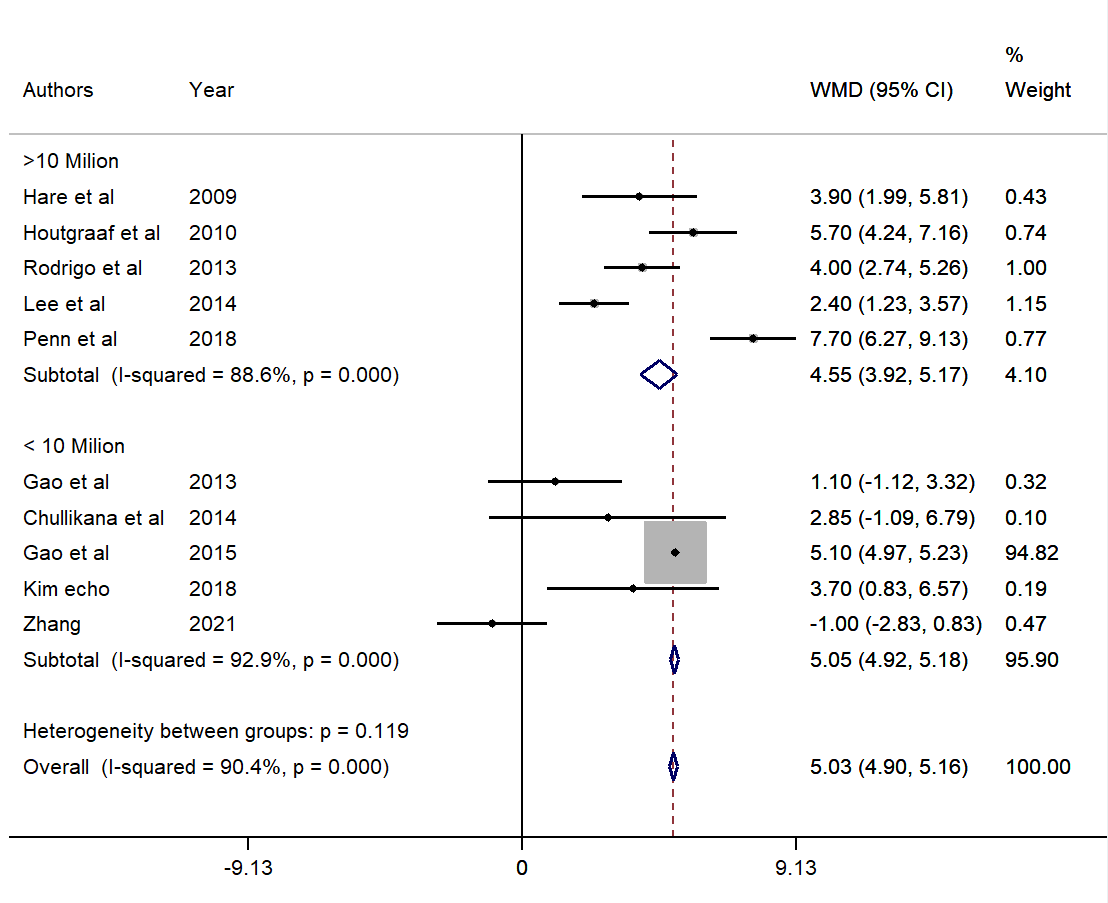

Supplement: Supplementary file 5 — Additional file 5. Subgroup analysis for the number of cells transplanted before and after excluding the biased studies. [file 13287_2021_2667_MOESM5_ESM.tif]

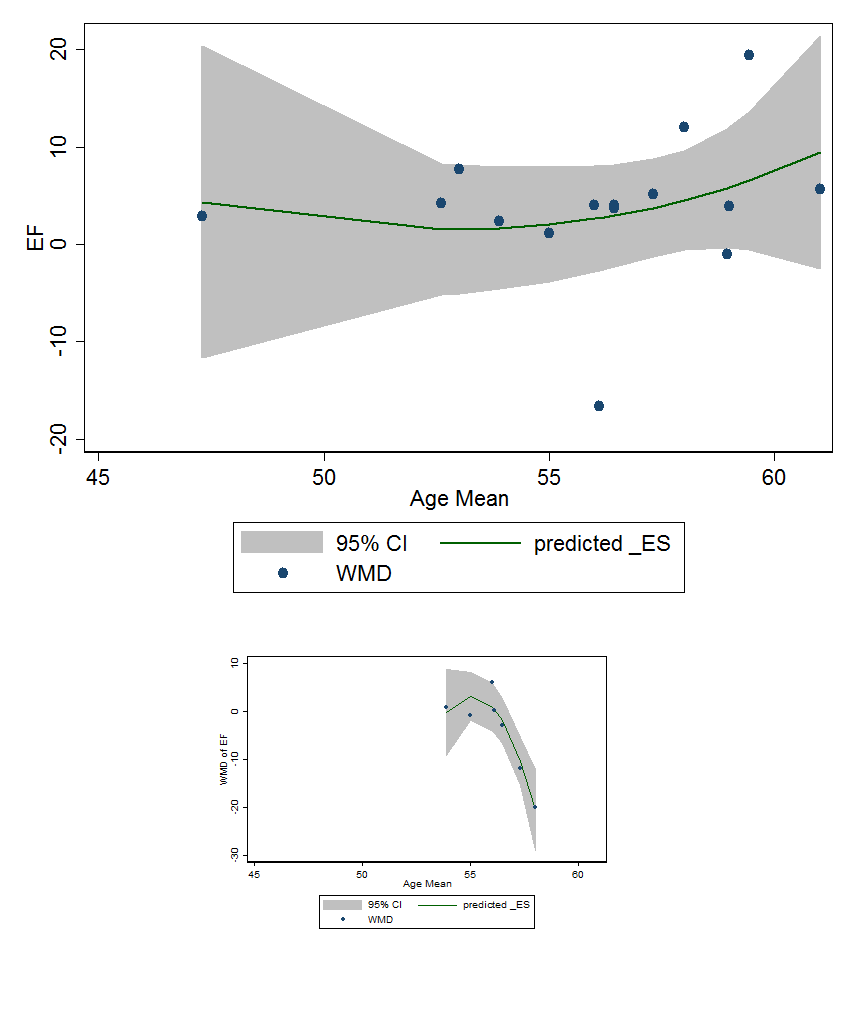

Supplement: Supplementary file 6 — Additional file 6. Dose response analysis for age before (top) and after (Bottom) excluding the biased studies. [file 13287_2021_2667_MOESM6_ESM.tiff]

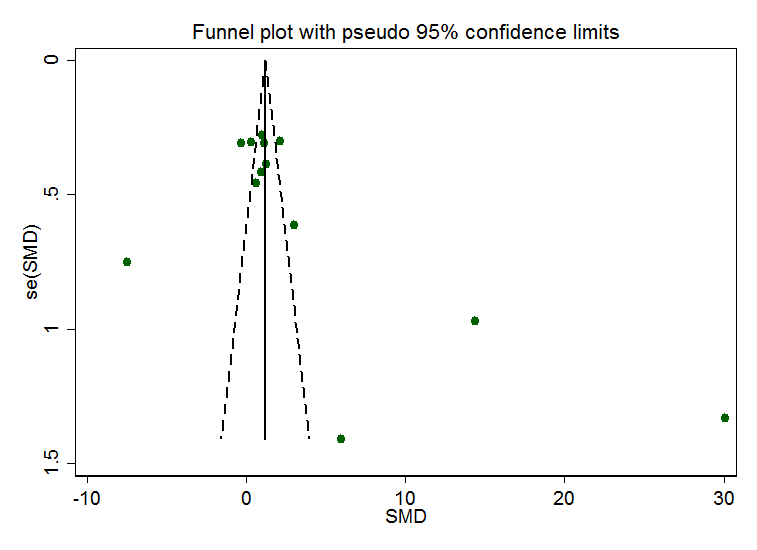

Supplement: Supplementary file 7 — Additional file 7. Publication bias analysis. [file 13287_2021_2667_MOESM7_ESM.tif]
